# The Global Atlas of Bamboo and Rattan (GABR) Phase II: new resources for sustainable development

**DOI:** 10.1093/gigascience/giac113

**Published:** 2022-10-30

**Authors:** Hansheng Zhao, Yinguang Hou, Jian Wang, Lei Sun, Junwei Gan, Yufei Meng, Zhiqiang Li, Shanying Li, Zeyu Fan, Yu Wang, Benhua Fei

**Affiliations:** Key Laboratory of National Forestry and Grassland Administration/Beijing for Bamboo & Rattan Science and Technology, International Centre for Bamboo and Rattan, Beijing 100102, China; Key Laboratory of National Forestry and Grassland Administration/Beijing for Bamboo & Rattan Science and Technology, International Centre for Bamboo and Rattan, Beijing 100102, China; Key Laboratory of National Forestry and Grassland Administration/Beijing for Bamboo & Rattan Science and Technology, International Centre for Bamboo and Rattan, Beijing 100102, China; Key Laboratory of National Forestry and Grassland Administration/Beijing for Bamboo & Rattan Science and Technology, International Centre for Bamboo and Rattan, Beijing 100102, China; Key Laboratory of National Forestry and Grassland Administration/Beijing for Bamboo & Rattan Science and Technology, International Centre for Bamboo and Rattan, Beijing 100102, China; Key Laboratory of National Forestry and Grassland Administration/Beijing for Bamboo & Rattan Science and Technology, International Centre for Bamboo and Rattan, Beijing 100102, China; Key Laboratory of National Forestry and Grassland Administration/Beijing for Bamboo & Rattan Science and Technology, International Centre for Bamboo and Rattan, Beijing 100102, China; Key Laboratory of National Forestry and Grassland Administration/Beijing for Bamboo & Rattan Science and Technology, International Centre for Bamboo and Rattan, Beijing 100102, China; Key Laboratory of National Forestry and Grassland Administration/Beijing for Bamboo & Rattan Science and Technology, International Centre for Bamboo and Rattan, Beijing 100102, China; Key Laboratory of National Forestry and Grassland Administration/Beijing for Bamboo & Rattan Science and Technology, International Centre for Bamboo and Rattan, Beijing 100102, China; Key Laboratory of National Forestry and Grassland Administration/Beijing for Bamboo & Rattan Science and Technology, International Centre for Bamboo and Rattan, Beijing 100102, China

**Keywords:** Bamboo, Rattan, GABR, Phase I, Phase II

## Abstract

Bamboo, the fast-growing grass plant, and rattan, the spiky climbing palm, are both essential forest resources that have been closely linked with human lives, livelihoods and material culture since ancient times. To promote genetic and genomic research in bamboo and rattan, a comprehensive and coordinated international project, the Genome Atlas of Bamboo and Rattan (GABR), was launched in 2017. GABR achieved great success during Phase I (2017-2022). We will focus on investigating and protecting bamboo and rattan germplasm resources in Phase II ( 2022-2027). Here, we briefly review the achievements of Phase I and introduce the goals of Phase II.

## Introduction

There are 1,642 species of bamboo and 631 known species of rattan, each with very different properties and potential uses [1]. Bamboo and rattan resources are widely distributed worldwide, mainly in tropical and subtropical areas, and provide unique ecological, economic and cultural services. They can help solve a series of global challenges and play an important role in developing a green economy, addressing climate change, building disaster-resilient infrastructure, alleviating poverty, revitalising rural areas, and protecting the environment. In recent years, bamboo and rattan have become an essential part of the international sustainable development conversation as critical tools for promoting South-South cooperation, implementing China's Belt and Road initiative, and contributing to the United Nations 2030 Sustainable Development Goals. The bamboo and rattan trade is estimated at USD 60 billion per year by the International Bamboo and Rattan Organization (INBAR), with domestic trade accounting for the majority. International trade in bamboo and rattan products has been increasing rapidly, reaching USD 3.417 billion per year in 2019 [2]. The bamboo and rattan trade in China generated more than USD 45 billion per year in 2021 [3].

Humankind has reached a new era in understanding, utilizing, and conserving biodiversity due to remarkable advances in genome sequencing technology, informatics, automation, and artificial intelligence. Hence, we launched the Genome Atlas of Bamboo and Rattan (GABR) in 2017 [4–5], which aims to sequence most bamboo and rattan species. Exploring the secrets in their genomes will enable us to understand how they evolved, resulting in radical new approaches for combating climate change-related biodiversity loss, improving agriculture, developing a sustainable global economy, restoring ecosystems, preserving species, and preventing future pandemics. Since then, significant progress has been made in Phase I, as outlined in the article describing the project's organization, goals, and strategies [4]. The successful conclusion of Phase I (July 2017-June 2022) was followed by the inception of Phase II (July 2022-June 2027). Hundreds of global scientists and institutions are working together to collect, investigate, and sequence bamboo and rattan germplasm resources.

## Phase I achievements

**Figure 1:**
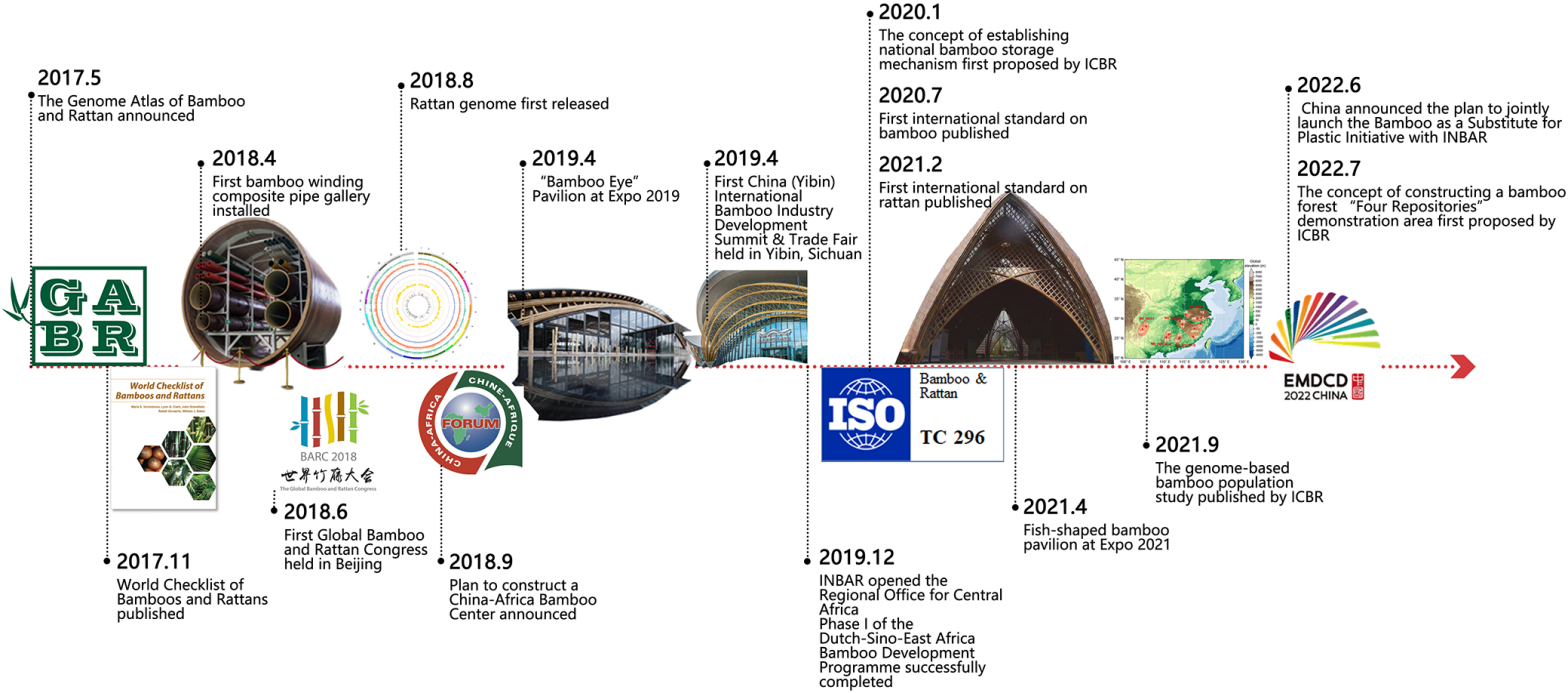
The significant events in the field of bamboo and rattan research ( 2017-2022). Further details are provided in Additional Table S7, with thanks to Dr Yufei Meng for the infographic.

Aided by the launch of the GABR project, in recent years significant achievements have been made in the field of bamboo and rattan research (Fig. 1). More than 3,500 transcriptome datasets and 800 genomic datasets have been released (Additional Table S1-4). Phase I targeted 2 subprojects and 11 representative topics, and after five years of smooth coordination of the project most of these targets have been completed and results published. The details of these have been provided in the checklist for target completion (Additional Table S5) and the project's website [5]. Among these achievements, one of the most significant has been the discovery of the low genetic diversity in moso bamboo [6]. Low genetic diversity can have severe effects on a species in terms of its survival and adaptation to the environment, as alongside far-reaching negative consequences such as decreased population adaptability and fewer opportunities for genetic improvement. Therefore, the conservation of bamboo genetic diversity is on the agenda going forward as an imperative strategic task for ensuring the bamboo industry's sustainable and healthy development.

Data collected from Phase I provided important basic information for developing new varieties and interpreting new phenotypes (such as *Phyllostachys edulis* cv. Pachyloen). The multi-omics studies in Phase I identified critical genes that were highly likely to contribute to important phenotypic traits and phenomena (such as fast-growing related genes, see Additional Table S6). These genes will be further validated *in vivo* to provide key candidate genes for the development of new bamboo and rattan varieties.

## Phase II goals

Based on the data and experience in Phase I, we are accelerating the expansion of our knowledge of bamboo and rattan. Phase II will conduct more in-depth, comprehensive and systematic investigations. The following four goals have been established.

- **Germplasm resources survey**. It will be necessary to conduct an extensive sequencing analysis of bamboo to characterize genome-level differences and variations among different species and areas. In Phase II, bamboo resources will be globally surveyed and collected from ∼100 regions around the world. Including ∼ 1,000 moso bamboo from different regions, alongside other species and ∼20 phenotypes (Additional Table S8). Resequencing and Genome-Wide Association Studies (GWAS) will be performed based on the ∼ 1,000 genomes to identify genes related to the cell wall. Additionally, pan-genomes will be built to reveal variants within genera or species.
- **High-quality reference genomes**. Phase II will cover about 30 species, with allele-aware chromosome-scale genomes of bamboo and rattan (Additional Table S8). A key objective is to select species with high economic value, excellent characteristics, and critical evolutionary positions.
- **Properties studies**. GABR will analyze the high-quality property of bamboo and rattan based on existing morphological structures, cell structure research, etc. GABR will also use state-of-the-art technologies (such as single-cell spatiotemporal transcriptome analysis) to identify different cell types and their functions.
- **Function verification**. GABR has long been committed to translating its theoretical achievements into practical applications. As breakthroughs have been made in bamboo transgenic technology, we can now verify candidate genes in bamboo and rattan *in vivo* and then apply these genes in genetic breeding and downstream production.

In terms of data sharing and consortium participation, Phase II will follow the open data policies previously made in the announcement paper [4]. Additionally, GABR continuously improves related policies to better serve the majority of scientific researchers.

## BARC facilitates GABR's development

INBAR is an intergovernmental development organization with 48 member states. To promote environmentally sustainable development using bamboo and rattan, INBAR co-initiated GABR and also co-hosts the Global Bamboo and Rattan Congress (BARC). The BARC provides a routine platform for worldwide participants to discuss the development of bamboo and rattan. It also promotes bamboo and rattan's significant role in global sustainable development. The first BARC was held in June 2018 under the theme of “enhancing South-South Cooperation for green development through bamboo and rattan's contribution to the sustainable development goals.” In November 2022, the second BARC is being held under the theme “nature-based solutions for sustainable development.” The Congress includes dialogues with high-level policymakers, product exhibitions and parallel sessions (including GABR side-events). Further information is provided on the event website [7].

## Conclusions

As the start-up phase of GABR, the past 5 years have seen significant progress, with the successful completion of Phase I goals. This project has ignited a tremendous amount of passion and energy among its participants, particularly the younger generation of scientists and the general public. However, Phase II presents many significant challenges, as does the precarious state of bamboo and rattan biodiversity; a coordinated effort across many institutions and scientists is therefore required. Let us move forward with GABR.

## Additional Files

**Additional Table S1**. Bamboo and rattan genomes published

**Additional Table S2**. Bamboo chloroplast genomes published

**Additional Table S3**. Bamboo transcriptomes published

**Additional Table S4**. Rattan transcriptomes published

**Additional Table S5**. A checklist for target completion

**Additional Table S6**. A list of fast-growing related genes in moso bamboo

**Additional Table S7**. The significant events in the field of bamboo and rattan research ( 2017-2022)

**Additional Table S8**. Target species in Phase II

**Additional Table S9**. Most of the funds for the project

giac113_GIGA-D-22-00260_Original_Submission

giac113_GIGA-D-22-00260_Revision_1

giac113_Response_to_Reviewer_Comments_Original_Submission

giac113_Reviewer_1_Report_Original_Submission Mingbing Zhou -- 10/10/2022 Reviewed

giac113_Reviewer_2_Report_Original_Submission Liang Wang -- 10/12/2022 Reviewed

giac113_Supplemental_File

## Data Availability

A detailed description of the project outputs can be found on the project's website [5] and the websites of the lead institutions (INBAR [8] and ICBR [9]). With data released via public databases including NCBI SRA and China National GeneBank (CNGB).

## Abbreviations

BARC: Global Bamboo and Rattan Congress; GABR: Genome Atlas of Bamboo and Rattan; GWAS: Genome-Wide Association Studies; INBAR: International Bamboo and Rattan Organization.

## Competing Interests

The authors declare that they have no competing interests.

## Author's contributions

H.S.Z., Y.G.H., J.W., Z.Q.L., and B.H.F. drafted the original manuscript text with detailed input from other authors. Y.F.M. drew the figure. All authors participated in the GABR project and have read and approved the final manuscript.

## Funding

This work was supported by the National Key Research and Development Program of China (2021YFD2201000) and the National Natural Science Foundation of China (31971733 and 31400557). Details on the funding for the project are provided in Additional Table S9.
