# Supplementary material for: The Global Atlas of Bamboo and Rattan (GABR) Phase II: new resources for sustainable development
Source: Gigascience. 2022 Oct 30;11:giac113. doi: 10.1093/gigascience/giac113 (PMC9618405; doi:10.1093/gigascience/giac113)

GigaScience

Bamboo and rattan's role in sustainable development will continue to be highlighted in Phase II of the Genome Atlas of Bamboo and Rattan (GABR) project

--Manuscript Draft--

Manuscript Number:	GIGA-D-22-00260
Full Title:	Bamboo and rattan's role in sustainable development will continue to be highlighted in Phase II of the Genome Atlas of Bamboo and Rattan (GABR) project
Article Type:	Commentary
Funding Information:	National Key Research and Development Program of China (2021YFD2201000) — Prof. Hansheng Zhao<br>National Natural Science Foundation of China (31971733) — Prof. Hansheng Zhao<br>National Natural Science Foundation of China (31400557) — Prof. Hansheng Zhao
Abstract:	Bamboo, the fast-growing grass plant, and rattan, the spiky climbing palm, are both essential forest resources that have been closely linked with human lives, livelihoods and the material culture since ancient times. To promote genetic and genomic research in bamboo and rattan, a comprehensive and coordinated international project, the Genome Atlas of Bamboo and Rattan (GABR), was launched in 2017. GABR achieved great success during Phase I (2017-2022). We will focus on investigating and protecting bamboo and rattan germplasm resources in Phase II. Additionally, the Second Global Bamboo and Rattan Congress (BARC 2022) will be held in Beijing on 7-8 November 2022. Here we briefly review Phase I, preview Phase II, and introduce the upcoming BARC 2022.
Corresponding Author:	Hansheng Zhao<br>International Center for Bamboo and Rattan<br>Beijing, Beijing CHINA
Corresponding Author Secondary Information:	
Corresponding Author's Institution:	International Center for Bamboo and Rattan
Corresponding Author's Secondary Institution:	
First Author:	Hansheng Zhao
First Author Secondary Information:	
Order of Authors:	Hansheng Zhao
	Jian Wang
	Yufei Meng
	Zhiqiang Li
	Shanying Li
	Zeyu Fan
	Junwei Gan
	Lei Sun
	Yinguang Hou
	Yu Wang
	Benhua Fei

Powered by Editorial Manager® and ProduXion Manager® from Aries Systems Corporation

Order of Authors Secondary Information:	
Additional Information:	
Question	Response
Are you submitting this manuscript to a special series or article collection?	No
**Experimental design and statistics**<br><br>Full details of the experimental design and statistical methods used should be given in the Methods section, as detailed in our Minimum Standards Reporting Checklist. Information essential to interpreting the data presented should be made available in the figure legends.<br><br>Have you included all the information requested in your manuscript?	Yes
**Resources**<br><br>A description of all resources used, including antibodies, cell lines, animals and software tools, with enough information to allow them to be uniquely identified, should be included in the Methods section. Authors are strongly encouraged to cite Research Resource Identifiers (RRIDs) for antibodies, model organisms and tools, where possible.<br><br>Have you included the information requested as detailed in our Minimum Standards Reporting Checklist?	Yes
**Availability of data and materials**<br><br>All datasets and code on which the conclusions of the paper rely must be either included in your submission or deposited in publicly available repositories (where available and ethically appropriate), referencing such data using a unique identifier in the references and in the "Availability of Data and Materials" section of your manuscript.	Yes

Powered by Editorial Manager® and ProduXion Manager® from Aries Systems Corporation

Have you have met the above requirement as detailed in our Minimum Standards Reporting Checklist?	

Manuscript

Click here to access/download;Manuscript;Commentary2022-v1.2.docx

Commentary

Bamboo and rattan's role in sustainable development will continue to be highlighted in Phase II of the Genome Atlas of Bamboo and Rattan (GABR) project

Hansheng Zhao, Jian Wang, Yufei Meng, Zhiqiang Li, Shanying Li, Zeyu Fan, Junwei Gan, Lei Sun, Yinguang Hou, Yu Wang, and Benhua Fei*

* Correspondence: feibenhua@icbr.ac.cn

Key Laboratory of National Forestry and Grassland Administration/Beijing for Bamboo & Rattan Science and Technology, International Centre for Bamboo and Rattan, Beijing 100102, China

Abstract

Bamboo, the fast-growing grass plant, and rattan, the spiky climbing palm, are both essential forest resources that have been closely linked with human lives, livelihoods and the material culture since ancient times. To promote genetic and genomic research in bamboo and rattan, a comprehensive and coordinated international project, the Genome Atlas of Bamboo and Rattan (GABR), was launched in 2017. GABR achieved great success during Phase I (2017-2022). We will focus on investigating and protecting bamboo and rattan germplasm resources in Phase II. Additionally, the Second Global Bamboo and Rattan Congress (BARC 2022) will be held in Beijing on 7-8 November 2022. Here we briefly review Phase I, preview Phase II, and introduce the upcoming BARC 2022.

Keywords: GABR, bamboo, rattan, BARC

Introduction

There are 1642 species of bamboo and 631 known species of rattan, each with very different properties and potential uses [1]. Bamboo and rattan resources are widely distributed worldwide, mainly in tropical and subtropical areas, and provide unique ecological, economic and cultural services. They can help solve a series of global challenges and play an important role in developing a green economy, addressing climate change, building disaster-resilient infrastructure, alleviating poverty, revitalising rural areas, and protecting the environment. In recent years, bamboo and rattan have become an essential part of the international sustainable development conversation as critical tools for promoting South-South cooperation, implementing China's Belt and Road initiative, and contributing to the United Nations 2030 Sustainable Development Goals. According to data from the United Nations Comtrade database, in 2019, exports of bamboo and rattan commodities reached USD 3.417 billion [2].

Humankind has reached a new era in understanding, utilizing, and conserving biodiversity due to remarkable advances in genome sequencing technology, informatics, automation, and artificial intelligence. Hence, we launched the Genome Atlas of Bamboo and Rattan (GABR) in 2017 [3], which aims to sequence most bamboo and rattan species. Exploring the secrets in their genomes will enable us to understand how they evolved, resulting in radical new approaches for combating climate change-related biodiversity loss, improving agriculture, developing a sustainable global economy, restoring ecosystems, preserving species, and preventing future pandemics. Since then, significant progress has been made in Phase I, as outlined in the article describing the project's organization, goals, and strategies [3]. The successful conclusion of Phase I was followed by the inception

of Phase II. Hundreds of scientists and institutions worldwide are working together to collect, investigate, and sequence bamboo and rattan germplasm resources globally.

Significant achievements in Phase I and the field of bamboo and rattan.

The past 5 years represent the start-up phase of GABR. To run the project smoothly, we started with building an international GABR network, including developing standards, evaluating strategies for producing reference genomes, and building communities. Subsequently, many advances have been made in research, including sequencing two rattan genomes [4], updating the bamboo genome [5], and identifying several essential functional genes[6-8]. Significant progress has been made in identifying a representative bamboo species, moso bamboo, with low genetic diversity [9]. Low genetic diversity can have severe consequences for species' survival and ability to adapt to the environment. It will also have highly far-reaching negative effects, including the loss of genes, reduced population adaptability, and little space for genetic improvement. Thus, the protection of bamboo genetic diversity is on the agenda, as an important strategic task for ensuring the bamboo industry's sustainable and healthy development.

Significant achievements made in the field of bamboo and rattan were summarized (Fig.1).

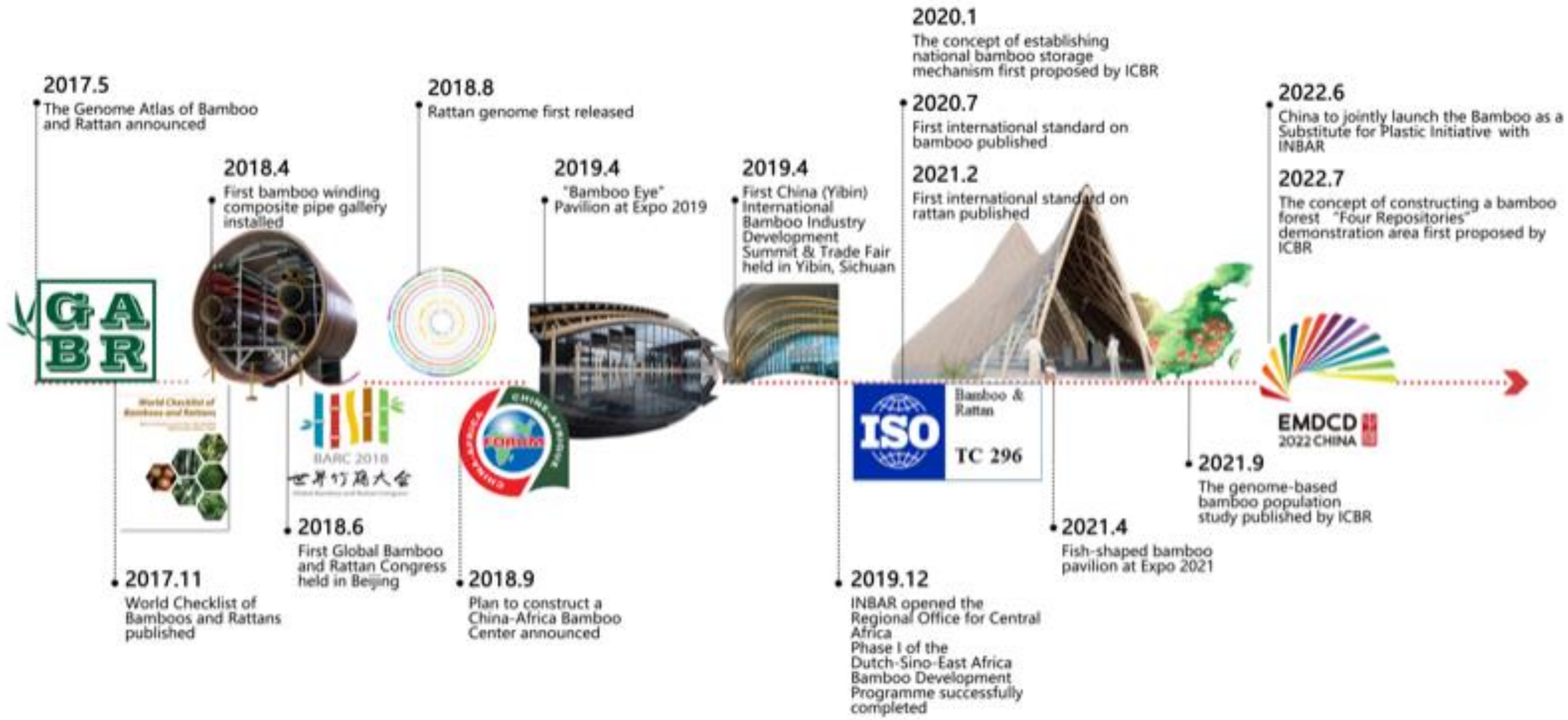

Fig. 1. The significant events in the field of bamboo and rattan (2017-2022). The details were provided in Additional file 1. The picture was drawn by Yufei Meng.

Progress toward GABR Phase II goals

Phase II of GABR will focus on resource collection and assessment in light of the experience gathered in Phase I. The year 2020 will be remembered as the beginning of the COVID-19 pandemic. Consequently, international cooperation in the study and collection of germplasm resources has been seriously affected. Fortunately, we were able to identify the genetic characteristics of bamboo populations based on the information provided by moso bamboo. Nevertheless, different species will have different features regarding their population genetics. Therefore, in Phase II, we will sequence more than 200 bamboo and rattan genomes, construct species- and genus-level pan-genomes and conduct the most recent survey of germplasm resources worldwide. In addition, we plan to conduct a single-cell spatiotemporal transcriptome analysis of bamboo and rattan using the latest omics and imaging techniques to identify different types of cells and their roles in bamboo formation.

The Second Global Bamboo and Rattan Congress (BARC 2022) will be held.

The Second Global Bamboo and Rattan Congress (BARC 2022) will be held in Beijing on 7-8 November 2022. The Congress will bring together people from across the world to discuss the development of bamboo and rattan and promote bamboo and rattan's important role in global sustainable development. BARC 2022 will be co-hosted by the International Bamboo and Rattan Organization (INBAR) and the National Forestry and Grassland Administration of China (NFGA) and co-organized by INBAR and the International Centre for Bamboo and Rattan (ICBR) under the theme "Nature-based Solutions for Sustainable Development". The Congress will include dialogues with high-level policymakers, product exhibitions and parallel sessions centering around four thematic areas: the road to carbon neutrality, green

industry and economic recovery, innovative materials and market development, and working together for inclusive and green development.

BARC 2022 will build on the achievements of GABR Phase I and BARC 2018, an international meeting connecting stakeholders from over 70 countries, making steps forward to promote bamboo and rattan as critical nature-based solutions. BARC 2022 will host representatives from governments, research institutes, international and non-governmental organizations, the private sector, the media, and other stakeholders who wish to learn more about bamboo and rattan's potential to contribute to their work. For more information on BARC 2022, please visit the BARC 2022 event website [10].

Conclusions

The past 5 years have seen significant progress for the GABR project, with the achievement of Phase I goals. This project has ignited a tremendous amount of passion and energy among its participants, particularly the younger generation of scientists and the general public. However, Phase II presents many significant challenges, as does the precarious state of bamboo and rattan biodiversity; a coordinated effort across many institutions and scientists is therefore required. Let us move forward with GABR.

Additional files

Additional file 1. A list of the significant events in the field of bamboo and rattan (2017-2022)

Abbreviations

BARC: Global Bamboo and Rattan Congress; GABR: Genome Atlas of Bamboo and Rattan; ICBR: International Centre for Bamboo and Rattan; INBAR: International Bamboo and Rattan Organization; NFGA: National Forestry and Grassland Administration of China.

Acknowledgements

We acknowledge the GABR Consortium members, partners, advisors, and supporters who have helped the GABR project run smoothly.

Funding

This work was supported by the National Key Research and Development Program of China (2021YFD2201000) and the National Natural Science Foundation of China (31971733 and 31400557).

Competing interests

The authors declare that they have no competing interests.

Author's contributions

H.S.Z., J.W., Z.Q.L., and B.H.F. drafted the original manuscript text with detailed input from other authors. Y.F.M. drew the figure. All authors participated in the GABR project and have read and approved the final manuscript.

References

1. Vorontsova MS, Clark LG, Dransfield J, Govaerts R and Baker WJ. World Checklist of Bamboos and Rattans. INBAR Technical Report No. 37. 2017.

2. International Bamboo and Rattan Organization. Trade Overview 2019: Bamboo and Rattan Commodities in China. 2021.
3. Zhao H, Zhao S, Fei B, Liu H, Yang H, Dai H, et al. Announcing the Genome Atlas of Bamboo and Rattan (GABR) project: promoting research in evolution and in economically and ecologically beneficial plants. GigaScience. 2017;6 7:1-7. doi:10.1093/gigascience/gix046.
4. Zhao H, Wang S, Wang J, Chen C, Hao S, Chen L, et al. The chromosome-level genome assemblies of two rattans (*Calamus simplicifolius* and *Daemonorops jenkinsiana*). GigaScience. 2018;7 9 doi:10.1093/gigascience/giy097.
5. Zhao H, Gao Z, Wang L, Wang J, Wang S, Fei B, et al. Chromosome-level reference genome and alternative splicing atlas of moso bamboo (*Phyllostachys edulis*). GigaScience. 2018;7 10 doi:10.1093/gigascience/giy115.
6. Chen M, Guo L, Ramakrishnan M, Fei Z, Vinod KK, Ding Y, et al. Rapid growth of Moso bamboo (*Phyllostachys edulis*): Cellular roadmaps, transcriptome dynamics, and environmental factors. The Plant Cell. 2022; doi:10.1093/plcell/koac193.
7. Li Y, Zhang D, Zhang S, Lou Y, An X, Jiang Z, et al. Transcriptome and miRNAome analysis reveals components regulating tissue differentiation of bamboo shoots. PLANT PHYSIOLOGY. 2022;188 4:2182-98. doi:10.1093/plphys/kiac018.
8. Ma X, Zhao H, Yan H, Sheng M, Cao Y, Yang K, et al. Refinement of bamboo genome annotations through integrative analyses of transcriptomic and epigenomic data. Computational and Structural Biotechnology Journal. 2021;19:2708-18. doi:10.1016/j.csbj.2021.04.068.

9. Zhao H, Sun S, Ding Y, Wang Y, Yue X, Du X, et al. Analysis of 427 genomes reveals moso bamboo population structure and genetic basis of property traits. Nature Communications. 2021;12 1 doi:10.1038/s41467-021-25795-x.

10. The Second Global Bamboo and Rattan Congress (BARC 2022). Introduction to BARC 2022. https://www.inbar.int/event/barc2022/.Accessed 15 Sep 2022.

Fig.1

Click here to access/download;Figure;Fig.1-revised.tiff

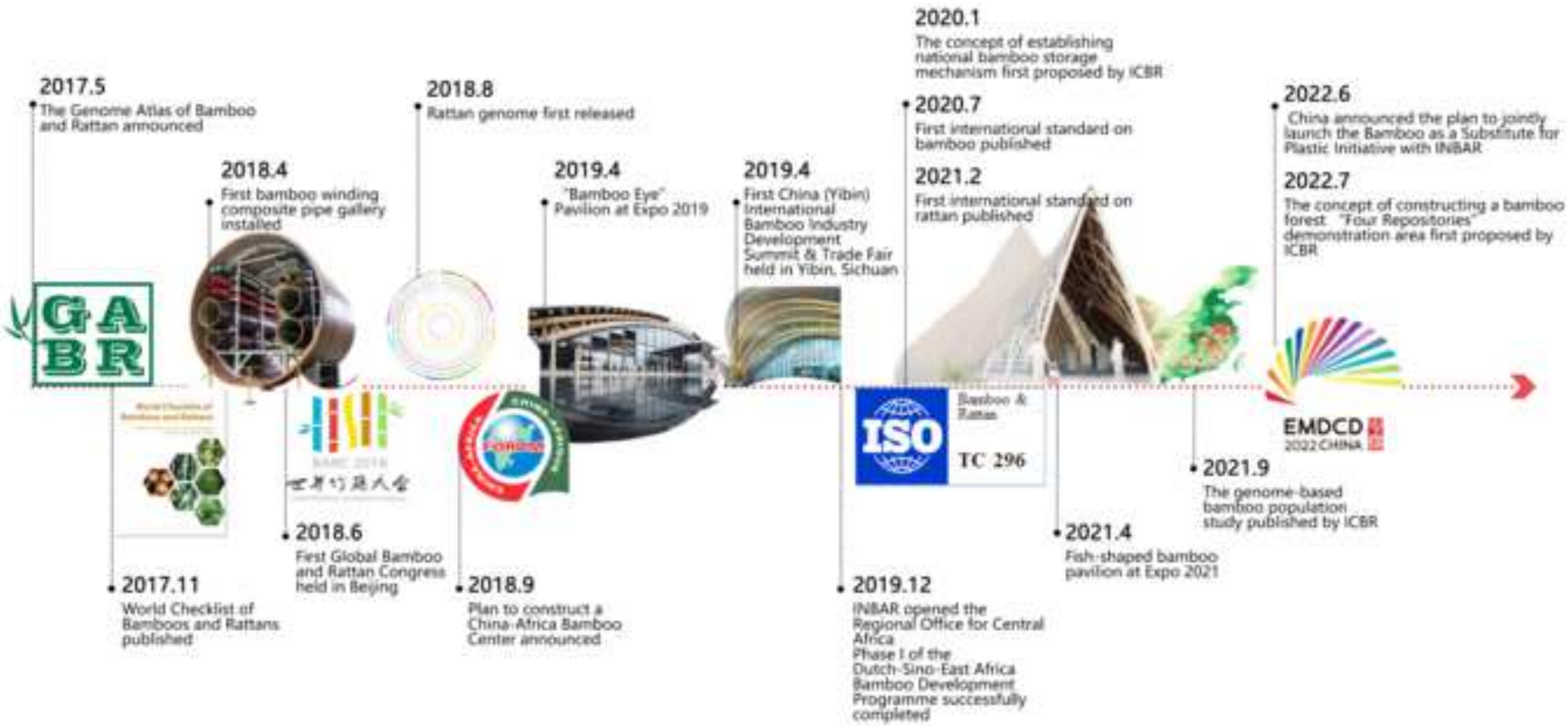

Supplement: giac113_GIGA-D-22-00260_Original_Submission [file giac113_giga-d-22-00260_original_submission.pdf]
