# Supplementary material for: The Global Atlas of Bamboo and Rattan (GABR) Phase II: new resources for sustainable development
Source: Gigascience. 2022 Oct 30;11:giac113. doi: 10.1093/gigascience/giac113 (PMC9618405; doi:10.1093/gigascience/giac113)
Supplement: giac113_Response_to_Reviewer_Comments_Original_Submission [file giac113_response_to_reviewer_comments_original_submission.pdf]

Author's Response To Reviewer Comments

Close

Responses to the comments of the Editor
1. Line 2-4: This is too long and not very focussed for a title and one of the reviewers suggests: "The Global Atlas of Bamboo and Rattan (GABR) Phase II: a fast-paced drive". Could also go for: "The Global Atlas of Bamboo and Rattan (GABR) Phase II: new resources for sustainable development"
Response:
Thanks for your suggestion. According to your suggestion, we have revised the title to "The Global Atlas of Bamboo and Rattan (GABR) Phase II: new resources for sustainable development"

2. Line21-22: Not sure its a good idea to promote a conference in a commentary, especially one that will be long finished by the time the paper is published and proofed. It certainly shouldn't be in the abstract.
Response:
Thanks for your suggestion. We have removed the related description, as follows:

"Here, we briefly review the achievements of Phase I and introduce the goals of Phase II"

3. Line53-54: Was there a specific time that Phase I ended and Phase II finished as might want to put the dates/significant event that marked this change?
Response:
Thanks for your suggestion. We have added the related description, as follows:

"The successful conclusion of Phase I (2017.7-2022.6) was followed by the inception of Phase II (2022.7-2027.6)."

4. Line58: In your announcement paper you announced projects, set targets, and talk about how to submit proposals. Did you achieve these? In particular what happened to the proposed website as that would be very useful to include here?
Response:
Thanks for your suggestion. We have added the related description, as follows:

"During the past few years, significant achievements have been made in the field of bamboo and rattan (Fig.1). More than 3500 transcriptome datasets and 800 genomic datasets have been released (Additional Table S1-4). Phase I targeted 2 subprojects and 11 representative topics. After five years of smooth running, most of these targets have been completed and results published. The details were provided in the checklist for target completion (Additional Table S5) and the project's website [5]."

5. Line 62-67: Any examples yet of people using this data in research or breeding programs? Interesting to hear about downstream reuse and wider achievements (standard development?) beyond citations of paper outputs.
Response:
Thanks for your suggestion. We have added the related descriptions, as follows:

"Data collected from Phase I provided important basic information for developing new varieties and interpreting new phenotypes (such as Phyllostachys edulis cv. Pachyloen). The multi-omics studies in Phase I identified critical genes that were highly likely to contribute to important phenotypic traits and phenomena (such as fast-growing related genes, see Additional Table S6). These genes will be further validated in vivo to provide key candidate genes for the development of new bamboo and rattan varieties."

6. Line 77-78: it should be "The significant events in the field of bamboo and rattan research (2017-2022). The details listed here are provided in more detail in Additional file 1. With thanks to Yufei Meng for the infographic."
Response:
Thanks for your suggestion. We have revised it accordingly, as follows:

“Fig. 1. The significant events in the field of bamboo and rattan research (2017-2022). Further details are provided in Additional Table S7, with thanks to Dr Yufei Meng for the infographic.”

7. Line90-91: Any info on how these are selected? Any way other bamboo and rattan researches can know what is coming, what they shouldn’t sequence, and what they need to do to join the consortium?
Response:
Thanks for your suggestion. According to your suggestion, we have rewritten the section and provided the species list in Phase I and Phase II (Additional Table S8).

8. Line93-95: In marker/announcement papers such as these it’s usually useful to say what the plans are for the data sharing too. In the announcement paper you had a section on this, and if you don’t want to repeat yourself you can tell readers can go back to that for more information (and for info on recruitment, etc.)
Response:
Thanks for your suggestion. We have added the related description, as follows:

“In terms of data sharing and consortium participation, Phase II will follow the policies previously made in the announcement [4]. Additionally, GABR continuously improves related policies to better serve the majority of scientific researchers. For example, some critical data can be shared with associated teams by signing an agreement beforehand.”

9. Line 97-119: OUP production takes at least one month so there is no way that this paper will be out before the meeting. Therefore this section is redundant and will be out of date. I would suggest cutting it, or at least massively reducing this and re-writing in the past rather than future tense. The Phase I and Phase II sections above are more interesting and more can be said in them.
Response:
Thanks for your suggestion. We have rewritten the section, as follows:

“BARC facilitates GABR's development
INBAR is an intergovernmental development organization with 48 member states. To promote environmentally sustainable development using bamboo and rattan, INBAR co-hosts the Global Bamboo and Rattan Congress (BARC) and co-initiated GABR. The BARC provides a routine platform for worldwide participants to discuss the development of bamboo and rattan. It also promotes bamboo and rattan’s significant role in global sustainable development. The first BARC was held in June 2018 under the theme of ‘enhancing South-South Cooperation for green development through bamboo and rattan’s contribution to the sustainable development goals’. In November 2022, the second BARC will be held under the theme ‘nature-based solutions for sustainable development’. The Congress includes dialogues with high-level policymakers, product exhibitions and parallel sessions (including GABR side-events). Further information was provided on the event website [7].”

10. All papers need this section now, and if there isn’t data you just say “Not applicable”, but in this case you can promote any portal or project IDs that phase I (and II) is going.
Response:
Thanks for your suggestion. We have added the related context, as follows:

“Data Availability
A detailed description of the project can be found on the project's website [5] or the website of the lead institutions (INBAR [8] and ICBR [9]). Most of the funds for the project were provided in Additional Table S9.”

Responses to the comments of Reviewer #1
Zhao et al. wrote the commentary "Bamboo and rattan's role in sustainable development will continue to be highlighted in Phase II of the Genome Atlas of Bamboo and Rattan (GABR) project". An overview of the achievements of GABR Phase I and the projects to be pursued in Phase II are presented in this article, as well as a description of the upcoming 2nd World Bamboo and Rattan Congress. To begin with, the article has updated a great deal of new statistical information in the field of bamboo and rattan, which is very useful for future scientific studies. The use of bamboo and rattan has become increasingly popular as a non-timber forest resource. Therefore, ICBR and other institutions launched the GABR program in 2017, which aims to advance scientific research in bamboo and rattan-related fields through

omics technology. The article briefly reviews the results of the first phase (2017-2022), including organizational management. Figure 1 was excellent because of
the exhibition of the progress of important bamboo and rattan fields. Based on the achievement of Phase I, Phase II is ready to collect and sequence large-scale germplasm resources for bamboo and rattan. The significance and scope of this work are enormous. Lastly, it is essential to hold the second World Bamboo and Rattan Congress to promote the development of the bamboo and rattan industries, strengthen communication, and enhance understanding. Despite an excellent job in the article writing, structure, and presentation, there are still some areas that could be improved, as follows:
1. The current title may be inappropriate for the commentary. It is suggested that the title be revised to "The Global Atlas of Bamboo and Rattan (GABR) Phase II: a fast-paced drive"
Response:
Thanks for your suggestion. We have revised it accordingly as follows:

“The Global Atlas of Bamboo and Rattan (GABR) Phase II: new resources for sustainable development”

2. The resolution of the "Fish-shaped bamboo pavilion at Expo 2021" in April 2021 in Fig. 1 is not high, and better high-resolution images are required.
Response:
Thanks for your suggestion. We have revised it accordingly.

3. Line16 "the" should be removed
Response:
Thanks for your suggestion. We have revised it accordingly.

4. Line52 revise to "global scientists and institutions"
Response:
Thanks for your suggestion. We have revised it accordingly.

5. Line63 revise to "a low"
Response:
Thanks for your suggestion. We have revised it accordingly.

6. Line64 revise to "severely affect"
Response:
Thanks for your suggestion. We have revised it accordingly.

7. Line70 revise to "in the bamboo and rattan field"
Response:
Thanks for your suggestion. We have revised it accordingly.

8. Line90 revise to"various features"
Response:
Thanks for your suggestion. We have revised it accordingly.

9. Line181 revise to "2182-2198"
Response:
Thanks for your suggestion. We have revised it accordingly.

10. Line186 revise to "2708-2718"
Response:
Thanks for your suggestion. We have revised it accordingly.

11. Line191 add a space in the front of the word Accessed
Response:
Thanks for your suggestion. We have revised it accordingly.

Responses to the comments of Reviewer #2
In this commentary, Zhao and colleagues reviewed the development history of GABR and several important scientific achievements in the field of bamboo and rattan during the past five years. Besides, the potential roles of bamboo and rattan in sustainable development, poverty reduction and climate change are also introduced. Finally, the authors outlooked the major mission of the Phase II of GABR. In

general, this commentary provides a comprehensive understanding of bamboo and rattan to both expert and non-specialized researchers.
However, I have several suggestions about this commentary.
1. The authors indicated that bamboo and rattan can help solve a series of global issues like climate change etc. These kinds of statements could be better supported by additional examples and data.
Response:
Thanks for your suggestion. According to your suggestion, we have added detailed data to support the statement that bamboo and rattan contribute significantly to the solution of global challenges, as follows:

"Bamboo and rattan trade is estimated at USD 60 billion by the International Bamboo and Rattan Organization (INBAR), with domestic trade accounting for the majority. International trade in bamboo and rattan products has been increasing rapidly, reaching USD 3.417 billion in 2019 [2]. The bamboo and rattan trade in China generated more than USD 45 billion in 2021 [3]."

2. The commentary would be benefited from adding additional genomic statistics data of bamboo and rattan, providing readers an intuitive understanding the genomic advance in bamboo and rattan.
Response:
Thanks for your suggestion. According to your suggestion, we have supplied related context as follows:

"During the past few years, significant achievements have been made in the field of bamboo and rattan (Fig.1). More than 3500 transcriptome datasets and 800 genomic datasets have been released (Additional Table S1-4). Phase I targeted 2 subprojects and 11 representative topics. After five years of smooth running, most of these targets have been completed and results published. The details were provided in the checklist for target completion (Additional Table S5) and the project's website [5]."

3. Details about the biological implication of low genetic diversity of bamboo are not necessary for a commentary, such as "Low genetic diversity can have severe consequences for species' survival and ability to adapt to the environment. It will also have highly far-reaching negative effects, including the loss of genes, reduced population adaptability, and little space for genetic improvement."
Response:
Thanks for your suggestion. According to your suggestion, we have revised it as follows:

"Low genetic diversity can have severe effects on a species in terms of its survival and adaptation to the environment, as well as having far-reaching negative consequences, such as decreased population adaptability and fewer opportunities for genetic improvement."

4. The resolution of the figure is needed to be improved.
Response:
Thanks for your suggestion. We have revised it accordingly.

5. The citation format for some references is needed to be revised and updated.
Response:
Thanks for your suggestion. We have revised it accordingly, as follows:

1. Vorontsova MS, Clark LG, Dransfield J, Govaerts R and Baker WJ. World Checklist of Bamboos and Rattans. INBAR Technical Report No. 37. 2017.
2. The International Bamboo and Rattan Organization. Trade Overview 2019: Bamboo and Rattan Commodities in the International Market. 2021.
3. China Forestry and Grassland. China foresty and grassland statistical yearbook of 2021. China Forestry Publishing House. 2021.
4. Zhao H, Zhao S, Fei B, Liu H, Yang H, Dai H, et al. Announcing the Genome Atlas of Bamboo and Rattan (GABR) project: promoting research in evolution and in economically and ecologically beneficial plants. GigaScience. 2017; 6(7):1-7. doi:10.1093/gigascience/gix046.
5. The GABR Project. http://www.gabr-project.com/. Accessed 10 Oct. 2022.
6. Zhao H, Sun S, Ding Y, Wang Y, Yue X, Du X, et al. Analysis of 427 genomes reveals moso bamboo population structure and genetic basis of property traits. Nature Communications. 2021; 12:1-12. doi:10.1038/s41467-021-25795-x.
7. The Second Global Bamboo and Rattan Congress. https://www.barc2022.inbar.int/#/home. Accessed 10 Oct. 2022.
8. International Bamboo and Rattan Organization. https://www.inbar.int/. Accessed 10 Oct. 2022.
9. International Centre for Bamboo and Rattan. http://eng.icbr.ac.cn/. Accessed 10 Oct. 2022.

Close
