# Supplementary material for: The Global Atlas of Bamboo and Rattan (GABR) Phase II: new resources for sustainable development
Source: Gigascience. 2022 Oct 30;11:giac113. doi: 10.1093/gigascience/giac113 (PMC9618405; doi:10.1093/gigascience/giac113)
Supplement: giac113_Reviewer_1_Report_Original_Submission — Mingbing Zhou -- 10/10/2022 Reviewed [file giac113_reviewer_1_report_original_submission.pdf]

Reviewer Report

Title: The Global Atlas of Bamboo and Rattan (GABR) Phase II: new resources for sustainable development

Version: Original Submission **Date:** 10/10/2022

Reviewer name: Mingbing Zhou

Reviewer Comments to Author:

Zhao et al. wrote the commentary "Bamboo and rattan's role in sustainable development will continue to be highlighted in Phase II of the Genome Atlas of Bamboo and Rattan (GABR) project". An overview of the achievements of GABR Phase I and the projects to be pursued in Phase II are presented in this article, as well as a description of the upcoming 2nd World Bamboo and Rattan Congress. To begin with, the article has updated a great deal of new statistical information in the field of bamboo and rattan, which is very useful for future scientific studies. The use of bamboo and rattan has become increasingly popular as a non-timber forest resource. Therefore, ICBR and other institutions launched the GABR program in 2017, which aims to advance scientific research in bamboo and rattan-related fields through omics technology. The article briefly reviews the results of the first phase (2017-2022), including organizational management. Figure 1 was excellent because of the exhibition of the progress of important bamboo and rattan fields. Based on the achievement of Phase I, Phase II is ready to collect and sequence large-scale germplasm resources for bamboo and rattan. The significance and scope of this work are enormous. Lastly, it is essential to hold the second World Bamboo and Rattan Congress to promote the development of the bamboo and rattan industries, strengthen communication, and enhance understanding. Despite an excellent job in the article writing, structure, and presentation, there are still some areas that could be improved, as follows:

1. The current title may be inappropriate for the commentary. It is suggested that the title be revised to "The Global Atlas of Bamboo and Rattan (GABR) Phase II: a fast-paced drive"
2. The resolution of the "Fish-shaped bamboo pavilion at Expo 2021" in April 2021 in Fig. 1 is not high, and better high-resolution images are required.
3. Line16 "the" should be removed
4. Line52 revise to "global scientists and institutions"
5. Line63 revise to "a low"
6. Line64 revise to "severely affect"
7. Line70 revise to "in the bamboo and rattan field"
8. Line90 revise to"various features"
9. Line181 revise to "2182-2198"
10. Line186 revise to "2708-2718"
11. Line191 add a space in the front of the word Accessed

Level of Interest

Please indicate how interesting you found the manuscript: Choose an item.

Quality of Written English

Please indicate the quality of language in the manuscript: Choose an item.

Declaration of Competing Interests

Please complete a declaration of competing interests, considering the following questions:

- Have you in the past five years received reimbursements, fees, funding, or salary from an organisation that may in any way gain or lose financially from the publication of this manuscript, either now or in the future?
- Do you hold any stocks or shares in an organisation that may in any way gain or lose financially from the publication of this manuscript, either now or in the future?
- Do you hold or are you currently applying for any patents relating to the content of the manuscript?
- Have you received reimbursements, fees, funding, or salary from an organization that holds or has applied for patents relating to the content of the manuscript?
- Do you have any other financial competing interests?
- Do you have any non-financial competing interests in relation to this paper?

If you can answer no to all of the above, write 'I declare that I have no competing interests' below. If your reply is yes to any, please give details below.

I declare that I have no competing interests.

I agree to the open peer review policy of the journal. I understand that my name will be included on my report to the authors and, if the manuscript is accepted for publication, my named report including any attachments I upload will be posted on the website along with the authors' responses. I agree for my report to be made available under an Open Access Creative Commons CC-BY license (http://creativecommons.org/licenses/by/4.0/). I understand that any comments which I do not wish to be included in my named report can be included as confidential comments to the editors, which will not be published.

Choose an item.

To further support our reviewers, we have joined with Publons, where you can gain additional credit to further highlight your hard work (see: https://publons.com/journal/530/gigascience). On publication of this paper, your review will be automatically added to Publons, you can then choose whether or not to claim your Publons credit. I understand this statement.

Yes Choose an item.
