# Supplementary material for: The Global Atlas of Bamboo and Rattan (GABR) Phase II: new resources for sustainable development
Source: Gigascience. 2022 Oct 30;11:giac113. doi: 10.1093/gigascience/giac113 (PMC9618405; doi:10.1093/gigascience/giac113)
Supplement: giac113_Reviewer_2_Report_Original_Submission — Liang Wang -- 10/12/2022 Reviewed [file giac113_reviewer_2_report_original_submission.pdf]

Reviewer Report

Title: The Global Atlas of Bamboo and Rattan (GABR) Phase II: new resources for sustainable development

Version: Original Submission **Date:** 10/12/2022

Reviewer name: Liang Wang

Reviewer Comments to Author:

In this commentary, Zhao and colleagues reviewed the development history of GABR and several important scientific achievements in the field of bamboo and rattan during the past five years. Besides, the potential roles of bamboo and rattan in sustainable development, poverty reduction and climate change are also introduced. Finally, the authors outlooked the major mission of the Phase II of GABR. In general, this commentary provides a comprehensive understanding of bamboo and rattan to both expert and non-specialized researchers.

However, I have several suggestions about this commentary.

1. The authors indicated that bamboo and rattan can help solve a series of global issues like climate change etc. These kinds of statements could be better supported by additional examples and data.
2. The commentary would be benefited from adding additional genomic statistics data of bamboo and rattan, providing readers an intuitive understanding the genomic advance in bamboo and rattan.
3. Details about the biological implication of low genetic diversity of bamboo are not necessary for a commentary, such as "Low genetic diversity can have severe consequences for species' survival and ability to adapt to the environment. It will also have highly far-reaching negative effects, including the loss of genes, reduced population adaptability, and little space for genetic improvement."
4. The resolution of the figure is needed to be improved.
5. The citation format for some references is needed to be revised and updated.

Level of Interest

Please indicate how interesting you found the manuscript: Choose an item.

Quality of Written English

Please indicate the quality of language in the manuscript: Choose an item.

Declaration of Competing Interests

Please complete a declaration of competing interests, considering the following questions:

- Have you in the past five years received reimbursements, fees, funding, or salary from an organisation that may in any way gain or lose financially from the publication of this manuscript, either now or in the future?
- Do you hold any stocks or shares in an organisation that may in any way gain or lose financially from the publication of this manuscript, either now or in the future?
- Do you hold or are you currently applying for any patents relating to the content of the manuscript?
- Have you received reimbursements, fees, funding, or salary from an organization that holds or has applied for patents relating to the content of the manuscript?
- Do you have any other financial competing interests?
- Do you have any non-financial competing interests in relation to this paper?

If you can answer no to all of the above, write 'I declare that I have no competing interests' below. If your reply is yes to any, please give details below.

I declare that I have no competing interests

I agree to the open peer review policy of the journal. I understand that my name will be included on my report to the authors and, if the manuscript is accepted for publication, my named report including any attachments I upload will be posted on the website along with the authors' responses. I agree for my report to be made available under an Open Access Creative Commons CC-BY license (http://creativecommons.org/licenses/by/4.0/). I understand that any comments which I do not wish to be included in my named report can be included as confidential comments to the editors, which will not be published.

Choose an item.

To further support our reviewers, we have joined with Publons, where you can gain additional credit to further highlight your hard work (see: https://publons.com/journal/530/gigascience). On publication of this paper, your review will be automatically added to Publons, you can then choose whether or not to claim your Publons credit. I understand this statement.

Yes Choose an item.
